# Quality of Life in Adult Individuals Living With or at Risk of a Hereditary Cancer Predisposition Syndrome: A Scoping Review of the Qualitative Literature

**DOI:** 10.1002/cam4.71069

**Published:** 2025-09-12

**Authors:** M. Sztankay, S. Wheelwright, V. Vassiliou, E. M. A. Bleiker, B. Rahman, B. Holzner, A. Yener, V. Engele, A. S. Oberguggenberger, EORTC Quality of Life Group

**Affiliations:** ^1^ Medical University of Innsbruck Innsbruck Austria; ^2^ University Hospital of Psychiatry II Innsbruck Austria; ^3^ Department of Clinical and Experimental Medicine, Brighton and Sussex Medical School University of Brighton and University of Sussex, Brighton UK; ^4^ Bank of Cyprus Oncology Centre, Radiation Oncology Department, Strovolos, Nicosia, Cyprus Strovolos Cyprus; ^5^ Department of Clinical Genetics, Leiden University Medical Center, Leiden, The Netherlands & Division of Psychosocial Research and Epidemiology & Department of Clinical Genetics, The Netherlands Cancer Institute Amsterdam Netherlands; ^6^ Department of Women's Cancer, EGA Institute for Women's Health, Faculty of Population Health Sciences University College London UK; ^7^ Portsmouth Hospitals University NHS Trust Southampton General Hospital Portsmouth UK

**Keywords:** disease susceptibility, genetic testing, health‐related quality of life, neoplasm, patient‐reported outcome, review

## Abstract

**Background:**

The in‐depth understanding of the impact of a hereditary cancer predisposition syndrome (HCPS) on the health‐related quality of life (HRQOL) of individuals with a hereditary cancer burden contributes to the improvement of counselling strategies as well as care planning and informs the development of patient‐reported outcome measures (PROMs) for standardised HRQOL assessment. This is the first review to systematically identify and synthesise the evidence from qualitative literature on HRQOL issues relevant for adult individuals living with (the risk of) HCPS between 1991 and 2024.

**Methods:**

Eligible studies were qualitative studies of adult individuals' experiences, including direct quotes and studies on the development or validation of health outcome measures. The literature was searched from 1991 to 2024 using the databases PubMed, CINAHL, Embase, and PsycINFO.

**Results:**

We screened 13,410 references for study inclusion by title and abstract, resulting in the retrieval of 606 full papers. More than 6800 qualitative patient quotes were extracted and coded by four raters. Reviewed literature provided a comprehensive picture of the experience of individuals living with (the risk of) HCPS in nine identified HRQOL domains (decision‐making, impact on family and social relationships, emotional response to test result, living with HCPS, perspective on life and self, HCPS‐related symptoms, taking measures to prevent the development or progression of cancer, issues related to the health care system, practical issues of life).

**Conclusion:**

Results contribute to insight on how individuals at risk cope with genetic testing and will inform the development of a PROM on their HRQOL that will be applicable for individualised patient management and service evaluation.

**Abbreviations**
EORTC: European Organisation for Research and Treatment of Cancer
GCT: genetic counselling and testing
HBOC: hereditary breast and ovarian cancer
HCPS: hereditary cancer predisposition syndrome
HRQOL: health‐related quality of life
PROM: patient‐reported outcome measure
QLG: quality of life group
VUS: variant of uncertain clinical significance

## 1 Introduction

With technology advancing, genetic counselling and testing (GCT) for the identification of hereditary pathogenic variations associated with increased cancer risk, commonly designated as Hereditary Cancer Predisposition Syndromes (HCPS), have become an integral part of medical oncology and are increasingly offered as a routine service in cancer hospitals for cancer patients and as (subsequent) predictive testing of their non‐affected relatives. This is attributable to the development of targeted therapies based on genetic markers and the recognised benefits of identifying unaffected at‐risk individuals, such as the possibility of providing prophylactic interventions to reduce the risk of developing cancer [1, 2, 3, 4]. Hence, the group of individuals known to be affected by or at risk for a hereditary cancer is constantly increasing [5] and referred to GCT. Genetic counselling is defined by the National Society of Genetic Counsellors in the United States as “the process of helping people understand and adapt to the medical, psychological and familial implications of genetic contributions to disease” [6]. Supporting counselees in adapting to potential implications necessitates not only an in‐depth understanding of the impact of an HCPS on the health‐related quality of life (HRQOL) of individuals with a hereditary cancer burden but also a systematic assessment of the latter. Herein, HRQOL refers to the impact of health, illness and treatment on a person's life as well as their functional status and ability to perform daily activities, which is generally assessed by patient‐reported outcome measures (PROMs) [7].

The experience of GCT and living with familial or hereditary cancer can affect individuals in a number of ways, influencing their HRQOL, health behaviour, and both family and peer support systems [8]. Although available evidence has illustrated that GCT is not associated with overall serious long‐term psychological morbidity, a quarter of counselees experience clinically relevant adverse psychological effects as well as HRQOL impairments [9, 10]. A (potentially) increased cancer lifetime risk, problems with perceived cancer control, or a test result of a genetic variant of uncertain clinical significance (VUS) can result in increased GCT‐related distress [11]. Intra‐ and interpersonal decisional conflicts such as individual distress or conflicts in the family, due to the increased complexity of the decision‐making process in the pre‐symptomatic setting, also have psychological implications such as anxiety and complex grief [12, 13].

Individuals with a known risk of an HCPS are advised to have regular screening for early detection of disease (e.g., mammography of breast and MRI for HBOC, colonoscopy for Lynch syndrome) or risk‐reducing treatment (e.g., oophorectomy and/or mastectomy for HBOC, gastrectomy for hereditary diffuse gastric cancer) [14, 15]. Preventive procedures entail hospital visits and a treatment‐related impact on HRQOL, especially in individuals at risk of developing multiple tumours and with certain risk factors, such as a personal history of cancer, having a first‐degree relative with cancer, negative illness perceptions, and coping styles [16]. At the same time, the knowledge of genetic predisposition can have positive implications in terms of self‐empowerment and increased social cohesion [17, 18].

GCT programs are constantly adapted to the rising requirements of clinical care and therefore need further elaborate measures to ensure comprehensive assessment of their impact on an individual's HRQOL, which can inform the overall management and well‐being of individuals living with or at risk of an HCPS. Even more so, as genetic testing is evolving from traditional testing models to contemporary models of mainstream and multigene panel testing [19]. However, established PROMs relating to HCPS are either disease‐ or counselee group‐specific [20] and focus on distinct aspects of HRQOL, such as cancer worry [21], coping [22] or satisfaction with counselling [23], limiting the comprehensive assessment of HRQOL. In this study, we systematically reviewed the qualitative literature in order to identify HRQOL issues relevant to individuals at risk of and/or already affected by an HCPS. This review is part of the development process of a European Organisation for Research and Treatment of Cancer Quality of Life Group (EORTC QLG) questionnaire for assessing the HRQOL of adult individuals at risk of or living with an HCPS.

## 2 Methods

Questionnaire development is being conducted according to the EORTC QLG guidelines for developing questionnaire modules, which follows four phases of questionnaire development [24], including the patient view as an essential source of information. The work reported herein refers to the identification of HRQOL issues based on the qualitative literature (part one of Phase I, including the structured extraction of direct patient quotes derived from qualitative studies for further evaluation of comprehensive content coverage). Qualitative studies are selected because they provide a more comprehensive insight into the counselees' perspective. Study reporting follows the Preferred Reporting Items for Systematic Reviews and Meta‐Analyses (PRISMA) guidelines [25].

### 2.1 Literature Search

The research question was: “What are the patient‐reported HRQOL issues for individuals with an HCPS or at risk for an HCPS?”. The original literature search (see Table S1) was conducted using the databases MEDLINE (PubMed), Web of Science Embase, CINAHL and PsycINFO, from 1991 to 2024. Keywords used in the literature search comprised [(Genetic predisposition AND Cancer & related diseases) OR HCPS OR Prophylactic surgical procedures] AND Quality of life. MeSH terms were combined with additional free text terms on HCPS derived from Garber & Offit [1].

Studies discussing any of the following were considered eligible: (1) qualitative studies providing quotes of patients' experiences; (2) development or validation of a health outcome measure; (3) use of a health outcome measure in a trial; and (4) any of the above if they included direct patient quotes. Health outcome refers to HRQOL, subjective health status, or psychological well‐being. Studies were only included if they contained direct patient quotes.

Eligibility criteria of the target population comprised the following: (1) adults diagnosed with an HCPS (with or without a cancer diagnosis); (2) adults who have a negative HCPS test result (i.e., are not identified as carriers of a pathogenic variant; with or without a cancer diagnosis); (3) adults with a test result of a VUS or familial cancer; or (4) adults who choose not to undergo testing despite being at risk of an HCPS. Overall, the target population comprised counselees at risk for any type of HCPS eligible for genetic testing (usually with a strong family history of cancer). Reviews or studies including children and adolescents (under the age of 18 years) were excluded.

#### 2.1.1 Screening Process

For the systematic literature review, a title and abstract screening was conducted by four independent reviewers (ASO, SW, MS, VE). Prior to the main screening phase, the reviewer team underwent a training process designed to calibrate their decision‐making and ensure consistency. In the pilot phase, all reviewers independently screened an initial set of 30 titles and abstracts. Discrepant ratings were discussed collectively to reach consensus and refine the screening criteria. This training procedure was repeated with new sets of titles and abstracts until a consensus rate of at least 90% was achieved. Following this calibration, the main screening phase commenced with all reviewers independently evaluating the remaining records, blinded to each other's evaluations. If both judges rated a search hit as eligible, the full papers were retrieved. The full papers were each reviewed by two different judges out of the reviewer team regarding eligibility. Disagreements were resolved by consensus discussion among the entire reviewer team. The screening procedure was performed by the use of EndNote and Excel.

#### 2.1.2 Coding Process

Qualitative content analysis was applied to identify recurring themes and patterns within the quotes, with reviewers coding the same content, based on an inductive approach, allowing categories to emerge directly from the data. The coding process followed a five‐step procedure, including familiarisation, code development, piloting, double coding, and resolution of discrepancies through discussion until consensus was reached. Multiple codes could be assigned to a single quote to capture the complexity of participants' experiences. Finally, individual codes were grouped into overarching content clusters to identify and organise key domains.

## 3 Results

**FIGURE 1:**
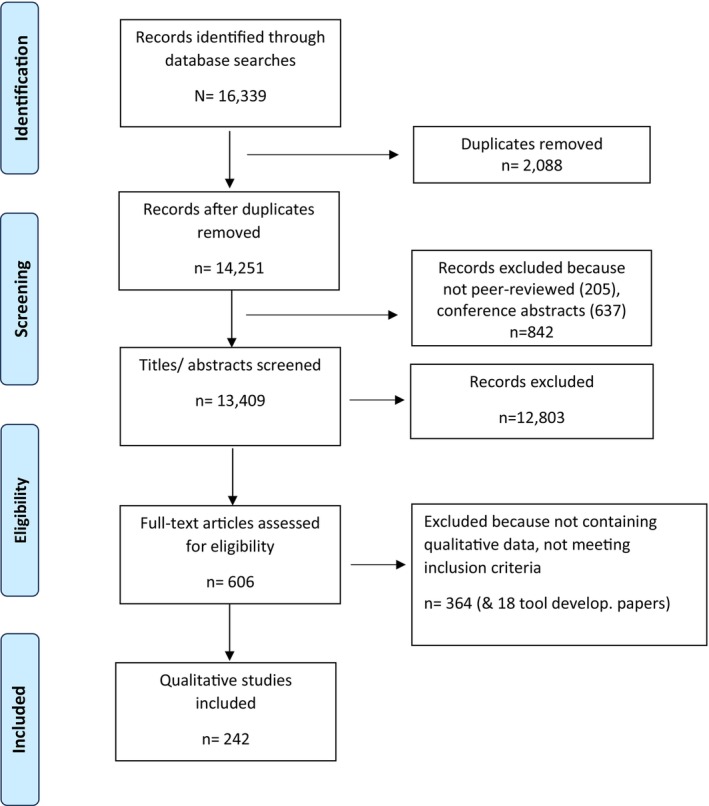
Literature search and selection procedure.

The literature search (see Figure 1) yielded 16.339 references containing one or more of the keywords. After the exclusion of duplicates and non‐peer‐reviewed articles, 13.410 references were screened for study inclusion, resulting in the retrieval of 606 full papers.

The characteristics of the 242 qualitative studies included are shown in summary Table 1 and in more detail in the online Table S2.

**TABLE 1:** Summary table for studies included (*N* = 242).

	*N* (%)
Age	
Range	18–83 years
Sex	
Female: male	83.3%:16.7%
Diverse	3 publications
HCPS	
HBOC	162 (66.9)
Multiple HCPS	28 (11.6)
HNPCC	11 (4.5)
Melanoma	6 (2.5)
FAP	5 (2.1)
Neurofibromatosis	5 (2.1)
Li Fraumeni	6 (2.5)
HGC	5 (2.1)
MEN	5 (2.1)
Other	5 (2.1)
Not reported	4 (1.7)
Country	
USA	82 (33.9)
UK	44 (18.2)
Australia	27 (11.2)
Canada	22 (9.1)
The Netherlands	12 (5.0)
Norway	5 (2.1)
France/Germany/Switzerland/Brasil	Each 4 (1.7)
Israel/Malaysia/Japan/Sweden/Denmark	Each 3 (1.2)
Ireland/Italy/New Zealand/Singapore	Each 2 (0.8)
Other	11 (4.5)
Methodology	
Cross‐sectional	211 (87.2)
Longitudinal	24 (9.9)
Pre‐post design	6 (2.5)
Other	1 (0.4)
Assessment via	
Interviews	202 (83.5)
Focus groups	17 (7.0)
Questionnaire (with free text)	14 (5.8)
Observation	7 (2.9)
Other	2 (0.8)
Genetic counselling	
Focus of study	51 (21.1)
Part of study	45 (18.6)
Endpoint of study	18 (7.4)

Abbreviations: FAB, familial adenomatous polyposis; HBOC, hereditary breast and ovarian cancer; HGC, hereditary gastric cancer; HNPCC, hereditary nonpolyposis colorectal cancer (incl. Lynch syndrome); MEN, multiple endocrine neoplasia.

Most of the studies were conducted in the United States (*n* = 82), United Kingdom (*n* = 44), Canada (*n* = 22) and Australia (*n* = 27) (see Table 1). The studies included interviews with 3 to 602 patients/participants, of whom 83.3% were female and 16.7% male, with an age range of 18 to 83 years. Some of the papers were reporting on the same interviews [26, 27]. Included studies addressed HBOC (66.9%), multiple HCPS (11.6%) and hereditary nonpolyposis colorectal cancer (4.5%). GC was the focus of (21.1%) or part of (18.6%) the studies included.

Each population group possibly affected by GCT was represented across the studies as a whole, and there were reports from the perspective of various groups for example male carriers of pathogenic variants or at risk, culture‐specific attributions to and perception of GCT by ethnic minorities, experience of non‐symptomatic carriers of pathogenic variants, living in an HCPS family as a non‐carrier, young and early adulthood, as well as from different settings, for example prophylactic or diagnostic.

One study reported on transgender patients' perspectives on their cancer genetic counselling experiences [28]. The most common thematic areas covered included the lived experience with HCPS (*n* = 48 studies), the impact of (referral to) GCT and the perception of GCT (*n* = 44), beliefs about personal risk and attitudes towards testing (*n* = 26), as well as the psychosocial impact of genetic result and HCPS (*n* = 26).

For a full list of the thematic foci and references of studies included, see Table S3.

The main source of qualitative data was interviews (83.5%) [29, 30], with focus groups (7%) [31, 32] and self‐administered patient questionnaires with free text response option (5.8%), making up the majority of the remainder. The remaining nine studies (3.7%) were based on observations and process analysis. There were a variety of qualitative methodologies employed (e.g., grounded theory, Interpretative Phenomenological Analysis). Study design was mainly cross‐sectional (87.2%).

### 3.1 Synthesis of HRQOL Issues

We extracted publications of the development of 18 tools [22, 33, 34], potentially containing qualitative data, and more than 6800 patient quotes.

Issues found were categorised into the following nine HRQOL domains: decision‐making, impact on family and social relationships, emotional response to test result, living with HCPS, perspective on life and self, HCPS‐related symptoms, taking measures to prevent the development or progression of cancer, issues related to the health care system and providers, and practical issues of life. The domains are illustrated with an exemplary patient quote in Table 2.

**TABLE 2:** HRQOL domains and example patient quotes from literature.

HRQOL domain	Domain description	Example patient quote
Decision‐making	Refers to the process individuals undergo when considering genetic testing and subsequent clinical actions	“*You know starting your family earlier, all those things that would have helped me plan my life a lot earlier […] so I just think it was more about me being proactive with my life choices*.” (woman tested negative for BRCA1 mutation, aged 24, [17])
Emotional response to test result	Refers to the wide range of emotional reactions shaped by the implications of the result, personal and familial cancer experiences and the perceived consequences of preventive options	“*I think I reacted in the grieving type process. At first, I was angry then progressed to acceptance then gathering information to my options*.” (woman tested positive for BRCA mutation after her breast cancer diagnosis, age unknown; [37])
Impact on family and social relationships	Refers to the emotional and communicative impact of HCPS on family dynamics, ranging from guilt and hesitation in sharing genetic information to strengthened bonds and empowerment through collective coping	“*[…] you are dealing with it sort of from a very personal point of view. When you go to Genetics, it's all of a sudden it's not just you anymore. It's the wider family and the implications for it and sort of not as much worried for myself then as for my own family and my brother and cousins and just the far reaching impact of what was happening to me. It was a different sensation completely from the diagnosis of cancer*.” (woman carrying BRCA mutation, age unknown [41])
Living with HCPS	Refers to the ongoing psychological and physical challenges linked to heightened cancer awareness and perceived mortality risk and the mitigating factors	“*[…] I don't think a day goes by that I don't think of it and sometimes thinking well what am I going to do about it? Or sometimes panicking, you know, what's going to happen if? A lot of things in terms of my daughter spring it to mind. Lots of things with the future you know make me a little bit apprehensive or bring it to mind anyway that I certainly carry this risk*.” (woman carrying BRCA mutation, age unknown [39])
Perspective on life and self	Refers to aspects of HCPS impacting counselees' identity and influencing body image, future planning and life choices	“*I have to honestly say that I feel somewhat betrayed by my body […]*. *Overall, I feel I am generally as healthy as I can be. But I don't trust that feeling and I don't trust my body. I have never put that in words before […] but it is so true*.” (non‐symptomatic woman carrying BRCAmutation for at least 4 years, age unknown [30])
		“*[*…*] if we had known that I was a carrier, then it is not for sure that we had been so [*…*] on that (to have children)*.” (wife of man carrying BRCA mutation, age unknown [27])
HCPS‐related symptoms	Refers to physical symptom burden and impaired functioning related to HCPS and its impact	“*Nf2 has totally changed my life and the lives of my immediate family. I can no longer go out to work due to the deafness, pain and other physical symptoms that I have*.” (female patient with neurofibromatosis, aged 54 [57])
Taking measures to prevent the development of progression of cancer	Refers to the impact of taking measures for risk reduction	“*It takes a lot to get over the fact that you've actually mutilated your own body. I looked at myself, thinking, ‘God what have I done to myself?’*” (woman carrying BRCA mutation after prophylactic mastectomy, age unknown [58])
		“*I feel that there are choices and options for the better about taking steps to prevent melanoma. It is not hopeless*.” (individual carrying CDKN2A/p16 mutation, age unknown [36])
		“*I mean like people say to me, ‘Oh, well, you know, I mean it can't get everybody in the family, you know.’ But I can't think like that. To me, it's not if, it's when*.” (woman carrying BRCA mutation, age unknown [12])
Issues related to health care system and providers	Refers to concerns about navigating the healthcare system	“*I just think that people will sit up and take more notice if I've had this testing. I'll get better treatment and, not necessarily better care, but better screening and everything I've mentioned before*.” (woman attending genetic clinic for prophylactic genetic testing for BRCA mutation, aged 24 [59])
Practical issues of life	Refers to the impact of HCPS on insurance, finances, employment, education and career decisions	“*Yes, obviously after these operations you will be on sick leave for quite some time, and then you will work part‐time, you are put into a position at work instead of being able to work full‐time and put your heart into something*.” (Swedish patient with multiple endocrine neoplasia type 1; sex and age unknown [60])

### 3.2 HRQOL Domains Based Across the GCT Trajectory

**FIGURE 2:**
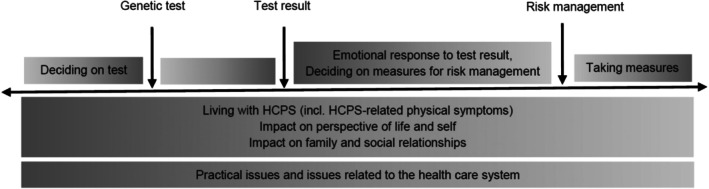
Model of the HRQOL domains across the GCT trajectory.

Patient quotes depicted the manifold ways HRQOL can be affected by facing and living with an HCPS, which is described in more detail below. Figure 2 shows a model of the different HRQOL domains across the GCT trajectory.

#### 3.2.1 Deciding on Taking the Test and on Measures of Risk Management

Deciding whether to undergo genetic testing for an HCPS and what clinical measures to take following a positive result is a central concern for many counselees. These decisions are often shaped by a combination of fear, perceived personal vulnerability, and a sense of responsibility towards family members. For some, providing information to descendants and informing their own oncological treatment (i.e., treatment‐focused GCT) plays a critical role in their decision‐making process:

I've got children so that makes a big difference to me. Um it's not just about me and my health and whether I get cancer and whether I die. It's my children and my family and how it's going to affect them. […] this is for their future as well. (woman prior to receiving genetic test result about carrying a BRCA mutation or not, aged 40 [35])

Predictive testing is often performed years before the likely onset of symptoms, which can contribute to emotional ambivalence—individuals may wish to know their risk while simultaneously fearing the consequences:

I mean like people say to me, ‘Oh, well, you know, I mean it can't get everybody in the family, you know.’ But I can't think like that. To me, it's not if, it's when. (woman with BRCA mutation prior to prophylactic ovariectomy, age unknown [12])

Certain groups, such as transgender individuals, may face additional barriers—such as disrupted family relationships—that limit their access to family health history and support. While current genetic counselling training emphasises the use of inclusive language and practices [24], recent findings suggest that transgender patients may still feel inadequately supported. This highlights a critical gap between training standards and patients' lived experiences, underscoring the need for ongoing efforts to ensure truly inclusive and affirming care.

#### 3.2.2 Emotional Response to the Test Result and to Risk‐Reducing Options

The genetic test result (positive, negative or of unknown variant) can cause relief and a feeling of empowerment since knowledge of risk enables taking action. It can also cause frustration about the possible lack of (preventive) treatment, for example CDKN2A/p16 mutation [36] or in transgender individuals due to potential impacts on gender affirmation therapies [28], and regret about not having been tested before the onset of symptoms.

I think I reacted in the grieving type process. At first, I was angry then progressed to acceptance then gathering information to my options. (woman tested positive for BRCA mutation after her breast cancer diagnosis, age unknown [37])

Past experiences with cancer in the family can be perceived as burdensome but also as reassuring due to having positive role models.

I had like three or four aunts die of cancer when I was really quite young. I remember that they were sick. I didn't really fully understand what cancer was but I knew that was why they were dying. And then, um, so, at a very young age we knew that there was cancer, a lot of cancer in our family. And then when my mom, um, got cancer, uh, then it was really close to home. My sister had cancer in her breasts … when she was 30 … And then my, more recently, my cousin passed away and it was, uh, she started with breast cancer and another cousin got breast cancer. And it was just everywhere. Everywhere we looked it was so prevalent. So [I've been] very aware of it since a very young age. (woman from a high risk family with known BRCA mutation, in her late 40s; [38])

The identification of a pathogenic variant provides options for early detection or prevention. Balancing perceived risk and anticipated consequences, the decision on making use of risk‐reducing options is a complex one. Counselees need to process the potential loss of functions due to prophylactic surgery (e.g., reproduction issues) which might be perceived as self‐harm bearing negative long‐term effects (e.g., on sex life, or on eating after prophylactic gastrectomy). On the other hand, preventive and screening options reduce anxiety and are reassuring with regard to risk reduction due to knowledge about predisposition. Having the opportunity to take precautions also provides the sense of self‐empowerment while seeking personal control of a (potential) cancer disease.

You know starting your family earlier, all those things that would have helped me plan my life a lot earlier […] so I just think it was more about me being proactive with my life choices. (woman tested negative for BRCA1 mutation, aged 24 [17])

#### 3.2.3 Living With HCPS and HCPS‐Related Symptoms

Living with an HCPS encompasses an increased awareness of signs of a possible cancer disease. The continuous confrontation with the threat of developing cancer or progression of disease and dying early might be accompanied by feelings of helplessness and guilt about transferring the risk to descendants.

[…] I don't think a day goes by that I don't think of it and sometimes thinking well what am I going to do about it? Or sometimes panicking, you know, what's going to happen if? A lot of things in terms of my daughter spring it to mind. Lots of things with the future you know make me a little bit apprehensive or bring it to mind anyway that I certainly carry this risk. (woman carrying BRCA mutation, aged 27, [39])

Psychological issues (e.g., feeling anxious, depressed) and physical problems due to HCPS differ according to the type of HCPS and can be very debilitating. The challenges of living with HCPS are more endurable when the family and peers provide practical as well as emotional support in terms of a sense of belonging. Role models originating from living in a family experienced with oncological diseases can further exert an impact on risk perception and choice of risk management.

[…] she was all right (mother) and like I say, I know my aunt was all right. So I'll be alright. (woman prior to prophylactic mastectomy, age unknown, [40])

#### 3.2.4 Impact on Family and Social Relationships

Quotes extracted from the literature highlighted the extended impact of HCPS from being on the individual to the wider social context with implications for family life and communication (e.g., avoiding talking about the effect of HCPS on daily life, being hesitant in disclosing information on genetic test results to children, different options on how to deal with HCPS).

[…] you are dealing with it sort of from a very personal point of view. When you go to Genetics, it's all of a sudden it's not just you anymore. It's the wider family and the implications for it and sort of not as much worried for myself then as for my own family and my brother and cousins and just the far reaching impact of what was happening to me. It was a different sensation completely from the diagnosis of cancer. (woman carrying BRCA mutation, age unknown; [41])

Guilt for passing on a pathogenic variant but also for taking the first steps towards genetic testing can aggravate reactions on the emotional level.

I was the first person in the family. I'm kind of the person who opened the can of worms. So, um, I felt that responsibility very heavily. (woman carrying BRCA mutation being the first in her family to attend GCT, age unknown [42])

Positive effects have also been reported such as feeling glad to be able to empower family members to take action, and the fact that dealing with HCPS together might strengthen emotional bonds and communication.

We stick together […] I think it is because we have been through so much. All those we have lost […] we know what they have been through. (woman with Lynch syndrome, age unknown [43])

#### 3.2.5 Impact on the Perspective on Life and Self

Being confronted with the finite nature of life due to HCPS may lead to changes in the perception of and the outlook on (future) life and self. Besides personal growth and a greater appreciation for and awareness of positive aspects, individuals report losing the notion of being healthy (i.e., now feeling like a patient) because of being affected by an HCPS. This evokes a feeling of being let down by one's body and increases distrust in the perception of oneself.

I have to honestly say that I feel somewhat betrayed by my body […]. Overall, I feel I am generally as healthy as I can be. But I don't trust that feeling and I don't trust my body. I have never put that in words before […] but it is so true. (non‐symptomatic woman carrying BRCA mutation for at least 4 years, age unknown, [30])

Reconstituting one's self‐concept includes dealing with changes in body image; for example, in women opting for prophylactic surgery, the worry about no longer being attractive to a potential partner.

[…] thought what does it mean to be a woman, our biology, the way we think and feel. I thought deeply about that, it would be crazy to keep ovaries and think I was more womanly if that would increase my chances to be around for children while they were growing up. Being a woman is more than just having ovaries. Women should consider their psychological state, consolidate sense of being a woman that transcends their view of physical biology. (woman carrying BRCA mutation after prophylactic oophorectomy, age unknown [44])

Making behavioural changes to reduce the chance of developing cancer (e.g., taking on a healthy lifestyle) goes hand in hand with concerns about how HCPS will affect life choices and future decisions on for example family planning. Disclosed genetic information by a parent with a pathogenic variant raises concerns about the future, especially regarding childbearing.

So that's putting a price, like a time limit, on when I can have kids. I just wanted to be an average 24‐year old girl. I didn't want to have to worry about having my kids early, having to have my life sped up from this one test result. To me, it's kind of like put a halt around my life. (daughter of a mother carrying BRCA mutation, age at testing unknown; [45])

Life choices might also be re‐evaluated in retrospect, for example wondering if one might have acted differently if the knowledge about an HCPS had been available earlier in life.

[…] if we had known that I was a carrier, then it is not for sure that we had been so […] on that (to have children). (wife of man carrying BRCA mutation, age unknown; [27])

#### 3.2.6 Practical Issues of Life and Issues Related to the Health Care System and Providers

Quotes referring to issues related to the health care system and providers illustrated counselees' attitudes towards GCT and the health care system. Besides being confident in available care, individuals might feel fraudulent about taking tests when not actually ill (i.e., non‐symptomatic).

I need somebody who can understand where I'm coming from and where I'm going to end up and who can support me. Above anything else is I need somebody who I know is not going to get terrified and run. (unaffected woman carrying BRCA mutation, aged 22, [46])

Practical issues of life include concerns about (getting or retaining) health insurance because of HCPS and negative effects on finances, employment (e.g., sick leave, taking time off for surgery), education and career choices as well as work relationships.

I was very stressed, couldn't sleep, so my working relationships were strained because of my stress. My boss suggested that I see a counselor to decide if I wanted to go through with the surgery or not […]. Afterwards, they all treated me like a cripple and were overprotective and thought I couldn't do anything. I had to keep reassuring them that I was ok, but they still treated me with kid gloves. It made me so angry. And then I gave up work, and because I wasn't working, I was strained financially. (female FAB patient, age unknown; [47])

## 4 Discussion

To the best of our knowledge, this is the first study to systematically identify and synthesise HRQOL aspects relevant to all groups of individuals living with or at risk for a HCPS. While many of the HRQOL themes identified are already familiar to the genetic counselling community, this review is unique in its systematic identification and synthesis of HRQOL issues across qualitative studies for all groups of potential counselees in GTC, including those often underrepresented in research in GTC, such as mutation‐negative individuals or unaffected relatives. This needs to be interpreted in the light of the fact that the majority of studies (83%) focused on female participants, only one specifically addressing the perceptions of transgender counselees [24] and were predominantly conducted in English‐speaking countries (72%).

The thematic diversity across the included studies reflects the complexity of living with an HCPS, yet generic HRQOL aspects emerged imminent to the hereditary risk of developing cancer. These include emotional responses to genetic testing, the impact on family dynamics, adaptation to perceived or real cancer risk, and challenges in navigating the healthcare system. The nine overarching HRQOL domains identified cut across different subgroups and echo findings from previous studies, indicating that individuals at genetic risk often face challenges similar to those living with chronic illness [48]. Furthermore, managing the response to hereditary cancer risk might be influenced more by the individual's emotional reactions and relational responsibilities than by rational considerations [49]. From the long‐term perspective, the knowledge of genetic susceptibility results in the recognition of the costs and benefits of this knowledge and the attempt to manage one's health in light of genetic risk [30]. The positive emotional impact of GCT might manifest in feelings of empowerment and social support from peers and the family, resulting in relief and reduction of anxiety.

### 4.1 Implications for Research and Practice

Considering HRQOL issues in clinical practice is vital for providing anticipatory guidance to potential behavioural and emotional responses to GCT and living with an HCPS. This includes the early identification of counselees whose risk of psychosocial morbidity might be underrated or who have unmet needs (e.g., descendants of carriers of pathogenic variants, mutation‐negative individuals living in affected families) and a targeted referral to expert support networks, including the uptake of multidisciplinary follow‐up if possible [50, 51]. Standardised PROMs are key for identifying and monitoring HRQOL issues systematically, supporting individualised care planning and ensuring that support structures are in place should an at‐risk individual later develop cancer.

As genetic testing expands beyond specialised settings, the need for validated PROMs becomes even more pressing [52, 53]. For this purpose, a routine, standardised assessment of PRO data (such as HRQOL) may foster further awareness and a more comprehensive detection of the varying demands and may contribute to the individualisation of patient management during the process of GCT and beyond [9], moving from recognition of HCPS‐related HRQOL issues to the systematisation of their assessment. Furthermore, routine application of standardised PROMs offers the opportunity to evaluate and refine genetic counselling services systematically, ensuring quality in a rapidly evolving field [54, 55]. Previous research demonstrated that the routine use of validated PRO questionnaires in clinical practice has a multitude of positive effects on both the process of care and patient outcomes [56].

### 4.2 Study Limitations

This review did not include a formal quality appraisal of the included qualitative studies, which may be seen as a limitation. However, our approach focused on original participant quotes rather than synthesised study results, which allowed for a direct analysis of HRQOL aspects in light of our research question. For this purpose, traditional methodological quality indicators such as trustworthiness or “research rigour” are of secondary importance.

In addition, females were overrepresented in the included studies, which may limit the generalizability of the findings to male counselees. However, this gender imbalance reflects current genetic counselling practice, where women are typically overrepresented due to gender‐specific cancer risks (i.e., HBOC) and higher uptake of genetic services. The former is also true for studies conducted in English‐speaking countries, as well as for underrepresented groups inadequately represented in research and with restricted healthcare access.

## 5 Conclusion

Living with or at risk for an HCPS affects multiple dimensions of HRQOL—not only for the individual, but often for the entire family system. While many of these aspects are recognised in genetic counselling practice and research, their consistent identification and management remain a challenge. Health professionals active in genetic counselling should be aware of these HRQOL aspects and make use of standardised PROMs to better capture and address patient needs throughout the genetic testing journey.

## Author Contributions

**M. Sztankay:** conceptualization (supporting), methodology (equal), formal analysis (lead), data curation (equal), writing – original draft (lead). **S. Wheelwright:** conceptualization (supporting), data curation (equal), formal analysis (supporting), writing – original draft (supporting), writing – review and editing (equal). **V. Vassiliou:** conceptualization (supporting), funding acquisition (equal), writing – review and editing (equal). **E. M. A. Bleiker:** conceptualization (supporting), writing – review and editing (equal). **B. Rahman:** writing – original draft (supporting), writing – review and editing (equal). **B. Holzner:** conceptualization (supporting), writing – original draft (supporting), writing – review and editing (equal). **A. Yener:** writing – original draft (supporting), writing – review and editing (equal). **V. Engele:** formal analysis (supporting), data curation (equal), writing – original draft (supporting), writing – review and editing (equal). **A. S. Oberguggenberger:** conceptualization (lead), funding acquisition (lead), methodology (equal), data curation (equal), formal analysis (lead), writing – original draft (supporting).

## Ethics Statement

The authors have nothing to report.

## Conflicts of Interest

The authors declare no conflicts of interest.

## Supporting information


**Table S1.** Example of search strategy and table of synonyms.

**Table S2.** Detailed characteristics of studies included.

**Table S3.** Thematic foci of studies included.

## Data Availability

The data used to support the findings of this study are included within the supporting information file.
