# Supplementary material for: Quality of Life in Adult Individuals Living With or at Risk of a Hereditary Cancer Predisposition Syndrome: A Scoping Review of the Qualitative Literature
Source: Cancer Med. 2025 Sep 12;14(18):e71069. doi: 10.1002/cam4.71069 (PMC12427361; doi:10.1002/cam4.71069)
Supplement: Supplementary file 1 — Table S1. Example of search strategy and table of synonyms. [file CAM4-14-e71069-s002.docx]

**Table S1.** Example of search strategy and table of synonyms (as online supplement)

|  | *Research question*: What are the quality of life issues for individuals with an HCPS or at risk for an HCPS?  *Search strategy*: [(Genetic predisposition AND Cancer & related diseases) OR HCPS OR Prophylactic surgical procedures] AND Quality of life | | |
| --- | --- | --- | --- |
| **Pubmed** | **Topic** | **MeSH terms** | **Additional free text** |
| **1** | Genetic predisposition | genetic predisposition to disease; genetic services | genetic predisposition, genetic susceptibility, genetic testing, genetic counse(l)ling, genetic screening |
| **2** | Cancer & related diseases | Neoplasms; Ataxia Telangiectasia; Rothmund-Thomson Syndrome; Bloom syndrome; Fanconi anemia; Nijmegen Breakage Syndrome; Wiskott-Aldrich Syndrome; Common Variable Immunodeficiency; Severe Combined Immunodeficiency; Lymphoproliferative Disorders; von  Hippel-Lindau Disease; Beckwith- Wiedemann syndrome | All known HCPS conditions (according to Table 2. [Hereditary Cancer](https://ascopubs.org/doi/10.1200/JCO.2005.10.042?url_ver=Z39.88-2003&rfr_id=ori%3Arid%3Acrossref.org&rfr_dat=cr_pub%20%200pubmed) [Predisposition Syndromes \| Journal of Clinical Oncology (ascopubs.org)](https://ascopubs.org/doi/10.1200/JCO.2005.10.042?url_ver=Z39.88-2003&rfr_id=ori%3Arid%3Acrossref.org&rfr_dat=cr_pub%20%200pubmed) |
| **3** | HCPS | neoplastic syndromes, hereditary; precancerous conditions; exostoses, multiple hereditary | Familial cancer |
| **4** | Prophylactic surgical procedures | prophylactic surgical procedures | Preventive surgical procedures |
| **5** | Quality of life | quality of life; patient outcome assessment; health status; self report; psychology; psychological adaptation; emotions | QOL, HRQOL, patient outcomes assessment, patient-centred outcomes research, subjective health status, patient reported outcome, patient based outcome, PRO, PROM, psychological well-being, psychological adjustment, psychological stress, cancer worry, disease worry |
| Within each topic, the Boolean operator OR was used for joining MeSH terms and additional free text together. | | | |

Medline Search

1. exp Genetic Predisposition to Disease/
2. exp Genetic Testing/
3. exp Genetic Counseling/
4. exp Neoplasms/
5. exp Ataxia Telangiectasia/
6. exp Rothmund-Thomson Syndrome/
7. exp Bloom Syndrome/
8. exp Fanconi Anemia/
9. exp Nijmegen Breakage Syndrome/
10. exp Wiskott-Aldrich Syndrome/
11. exp Common Variable Immunodeficiency/
12. exp Severe Combined Immunodeficiency/
13. exp Lymphoproliferative Disorders/
14. exp von Hippel-Lindau Disease/
15. exp Beckwith-Wiedemann Syndrome/
16. exp Neoplastic Syndromes, Hereditary/
17. exp Precancerous Conditions/
18. exp Exostoses, Multiple Hereditary/
19. exp "Quality of Life"/
20. exp Patient Outcome Assessment/
21. exp Health Status/
22. exp Self Report/
23. exp Psychology/
24. exp Adaptation, Psychological/
25. exp Emotions/
26. 1 or 2 or 3
27. 4 or 5 or 6 or 7 or 8 or 9 or 10 or 11 or 12 or 13 or 14 or 15
28. 16 or 17 or 18
29. 19 or 20 or 21 or 22 or 23 or 24 or 25
30. 26 and 27
31. 28 or 30

33. limit 32 to english language

32. 29 and 31
