# Supplementary material for: Quality of Life in Adult Individuals Living With or at Risk of a Hereditary Cancer Predisposition Syndrome: A Scoping Review of the Qualitative Literature
Source: Cancer Med. 2025 Sep 12;14(18):e71069. doi: 10.1002/cam4.71069 (PMC12427361; doi:10.1002/cam4.71069)
Supplement: Supplementary file 3 — Table S3. Thematic foci of studies included. [file CAM4-14-e71069-s003.docx]

| **Table S3.** Thematic foci of studies included (N=233) | | |
| --- | --- | --- |
|  | N | References |
| *lived experience with HCPS* | 48 | [1-48] |
| *adaptation to life transitions* | 8 | [49-56] |
| *beliefs about personal risk and attitudes towards testing* | 26 | [57-82] |
| *stigma* | 2 | [83, 84] |
| *responsibility of genetic risk* | 1 | [85] |
| *evaluating the decision-making process on genetic testing and risk-reducing options, including information and support need, barriers and*  *facilitators* | 41 | [86-126] |
| *psychosocial impact of genetic result and HCPS* | 26 | [127-152] |
| *impact of (referral to) GCT and perception of GCT* | 44 | [153-196] |
| *investigating the risk communication process* | 9 | [197-205] |
| *disclosing genetic result to children and other family members* | |  |
| *disclosing genetic results from the providing end* | 11 | [206-216] |
| *disclosing genetic results from the receiving end* | 4 | [217-220] |
| *impact of family history and experience of living in an HCPS-affected family* | 8 | [221-228] |
| *Expectations/ experiences regarding health care professionals or the health care system* | 5 | [229-233] |

1. Pasacreta JV: **Psychosocial issues associated with increased breast and ovarian cancer risk: findings from focus groups**. *Arch Psychiatr Nurs* 1999, **13**(3):127-136.
2. Giarelli E: **Bringing threat to the fore: participating in lifelong surveillance for genetic risk of cancer**. *Oncol Nurs Forum* 2003, **30**(6):945-955.
3. Hallowell N: **Varieties of suffering: Living with the risk of ovarian cancer**. *Health, Risk & Society* 2006, **8**(1):9-26.
4. Kenen R, Ardern-Jones A, Eeles R: **"Social separation" among women under 40 years of age diagnosed with breast cancer and carrying a BRCA1 or BRCA2 mutation**. *J Genet Couns* 2006, **15**(3):149-162.
5. Giarelli E: **Self-surveillance for genetic predisposition to cancer: behaviors and emotions**. *Oncol Nurs Forum* 2006, **33**(2):221-231.
6. Carlsson C, Nilbert M: **Living with hereditary non-polyposis colorectal cancer; experiences from and impact of genetic testing**. *J Genet Couns* 2007, **16**(6):811- 820.
7. Stromsvik N, Nordin K, Berglund G, Engebretsen LF, Hansson MG, Gjengedal E: **Living with multiple endocrine neoplasia type 1: decent care-insufficient medical and genetic information: a qualitative study of MEN 1 patients in a Swedish hospital**. *J Genet Couns* 2007, **16**(1):105-117.
8. Spector D: **Lifestyle behaviors in women with a BRCA1 or BRCA2 genetic mutation: an exploratory study guided by concepts derived from the Health Belief Model**. *Cancer Nurs* 2007, **30**(1):E1-10.
9. Bakos AD, Hutson SP, Loud JT, Peters JA, Giusti RM, Greene MH: **BRCA mutation- negative women from hereditary breast and ovarian cancer families: a**

**qualitative study of the BRCA-negative experience**. *Health Expect* 2008,

**11**(3):220-231.

1. Werner-Lin A: **Formal and informal support needs of young women with BRCA mutations**. *J Psychosoc Oncol* 2008, **26**(4):111-133.
2. Hoskins LM, Roy K, Peters JA, Loud JT, Greene MH: **Disclosure of Positive**. *Fam Syst Health* 2008, **26**(3):296-316.
3. Patenaude AF, Orozco S, Li X, Kaelin CM, Gadd M, Matory Y, Mayzel K, Roche CA, Smith BL, Farkas W *et al*: **Support needs and acceptability of psychological and peer consultation: attitudes of 108 women who had undergone or were considering prophylactic mastectomy**. *Psychooncology* 2008, **17**(8):831-843.
4. Hamilton R, Williams JK, Skirton H, Bowers BJ: **Living with genetic test results for hereditary breast and ovarian cancer**. *J Nurs Scholarsh* 2009, **41**(3):276-283.
5. Crump RJ, Fitzgerald RP, Legge M: **'Going-to-have-cancerness': a study of living with increased risk of BRCA1 and BRCA2 mutations for six South Island women**. *J Prim Health Care* 2010, **2**(4):311-317.
6. Bennett P, Parsons E, Brain K, Hood K, Team rS: **Long-term cohort study of women at intermediate risk of familial breast cancer: experiences of living at risk**. *Psychooncology* 2010, **19**(4):390-398.
7. Fritzell K, Persson C, Björk J, Hultcrantz R, Wettergren L: **Patients' views of surgery and surveillance for familial adenomatous polyposis**. *Cancer Nurs* 2010, **33**(2):E17-23.
8. Underhill ML, Dickerson SS: **Engaging in medical vigilance: understanding the personal meaning of breast surveillance**. *Oncol Nurs Forum* 2011, **38**(6):686-694.
9. Stromsvik N, Raheim M, Gjengedal E: **Cancer worry among Norwegian male BRCA1/2 mutation carriers**. *Fam Cancer* 2011, **10**(3):597-603.
10. Patel CM, Ferner R, Grunfeld EA: **A qualitative study of the impact of living with neurofibromatosis type 2**. *Psychol Health Med* 2011, **16**(1):19-28.
11. Underhill ML, Lally RM, Kiviniemi MT, Murekeyisoni C, Dickerson SS: **Living my family's story: identifying the lived experience in healthy women at risk for hereditary breast cancer**. *Cancer Nurs* 2012, **35**(6):493-504.
12. Persson E, Lindholm E, Berndtsson I, Lundstam U, Hultén L, Carlsson E: **Experiences of living with increased risk of developing colorectal and gynaecological cancer in individuals with no identified gene mutation**. *Scand J Caring Sci* 2012, **26**(1):20-27.
13. Hoskins LM, Roy KM, Greene MH: **Toward a new understanding of risk perception among young female BRCA1/2 "previvors"**. *Fam Syst Health* 2012, **30**(1):32-46.
14. Saleh M, Barlow-Stewart K, Meiser B, Tucker K, Eisenbruch M, Kirk J: **Knowledge, attitudes and beliefs of Arabic-Australians concerning cancer**. *Psychooncology* 2012, **21**(2):195-202.
15. DiMillo J, Samson A, Thériault A, Lowry S, Corsini L, Verma S, Tomiak E: **Living with the BRCA genetic mutation: an uncertain conclusion to an unending process**. *Psychol Health Med* 2013, **18**(2):125-134.
16. Petersen HV, Nilbert M, Bernstein I, Carlsson C: **Balancing life with an increased risk of cancer: lived experiences in healthy individuals with Lynch syndrome**. *J Genet Couns* 2014, **23**(5):778-784.
17. Jeffers L, Morrison PJ, McCaughan E, Fitzsimons D: **Maximising survival: the main concern of women with hereditary breast and ovarian cancer who undergo genetic testing for BRCA1/2**. *Eur J Oncol Nurs* 2014, **18**(4):411-418.
18. Samson A, DiMillo J, Thériault A, Lowry S, Corsini L, Verma S, Tomiak E: **Living with the BRCA1 and BRCA2 genetic mutation: learning how to adapt to a virtual chronic illness**. *Psychol Health Med* 2014, **19**(1):103-114.
19. Kasparian NA, Rutstein A, Sansom-Daly UM, Mireskandari S, Tyler J, Duffy J, Tucker KM: **Through the looking glass: an exploratory study of the lived experiences and unmet needs of families affected by Von Hippel-Lindau disease**. *Eur J Hum Genet* 2015, **23**(1):34-40.
20. Underhill M, Berry D, Dalton E, Schienda J, Syngal S: **Patient experiences living with pancreatic cancer risk**. *Hered Cancer Clin Pract* 2015, **13**(1):13.
21. Bicudo NP, de Menezes Neto BF, da Silva de Avó LR, Germano CM, Melo DG: **Quality of Life in Adults with Neurofibromatosis 1 in Brazil**. *J Genet Couns* 2016, **25**(5):1063-1074.
22. Dean M: **"It's not if I get cancer, it's when I get cancer": BRCA-positive patients' (un)certain health experiences regarding hereditary breast and ovarian cancer risk**. *Social Science & Medicine* 2016, **163**:21-27.
23. Dean M, Davidson LG: **Previvors' Uncertainty Management Strategies for Hereditary Breast and Ovarian Cancer**. *Health Communication* 2018, **33**(2):122- 130.
24. Etchegary H, Dicks E, Tamutis L, Dawson L: **Quality of life following prophylactic gynecological surgery: experiences of female Lynch mutation carriers**. *Familial Cancer* 2018, **17**(1):53-61.
25. Hamilton JG, Shuk E, Arniella G, Gonzalez CJ, Gold GS, Gany F, Robson ME, Hay JL: **Genetic Testing Awareness and Attitudes among Latinos: Exploring Shared Perceptions and Gender-Based Differences**. *Public Health Genomics* 2016, **19**(1):34-46.
26. Moynihan C, Bancroft EK, Mitra A, Ardern-Jones A, Castro E, Page EC, Eeles RA: **Ambiguity in a masculine world: Being a BRCA1/2 mutation carrier and a man with prostate cancer**. *Psycho-Oncology* 2017, **26**(11):1987-1993.
27. Myklebust M, Gjengedal E, Stromsvik N: **Experience of Norwegian Female BRCA1 and BRCA2 Mutation-Carrying Participants in Educational Support Groups: a Qualitative Study**. *Journal of Genetic Counseling* 2016, **25**(6):1198-1206.
28. Qiu J, Guan J, Yang X, Wu J, Liu G, Di G, Chen C, Hou Y, Han Q, Shen Z *et al*: **Quality of Life and Psychological State in Chinese Breast Cancer Patients Who Received BRCA1/2 Genetic Testing**. *PLoS ONE [Electronic Resource]* 2016, **11**(7):e0158531.
29. Rauscher EA, Dean M, Campbell-Salome G, Barbour JB: **'How do we rally around the one who was positive?' familial uncertainty management in the context of men managing BRCA-related cancer risks**. *Social Science & Medicine* 2019, **242**.
30. Rauscher EA, Dean M: **"I've just never gotten around to doing it": Men's approaches to managing BRCA-related cancer risks**. *Patient Education and Counseling* 2018, **101**(2):340-345.
31. Ross J, Bojadzieva J, Peterson S, Noblin SJ, Yzquierdo R, Askins M, Strong L: **The psychosocial effects of the Li-Fraumeni Education and Early Detection (LEAD) program on individuals with Li-Fraumeni syndrome**. *Genetics in Medicine* 2017, **19**(9):1064-1070.
32. Schroeder D, Duggleby W, Cameron BL: **Moving In and Out of the What-Ifs: The Experiences of Unaffected Women Living in Families Where a Breast Cancer 1 or 2 Genetic Mutation Was Not Found**. *Cancer Nursing* 2017, **40**(5):386-393.
33. Skop M, Lorentz J, Jassi M, Vesprini D, Einstein G: **"Guys Don't Have Breasts": The Lived Experience of Men Who Have BRCA Gene Mutations and Are at Risk for Male Breast Cancer**. *American Journal of Mens Health* 2018, **12**(4):961-972.
34. Getachew-Smith H, Ross AA, Scherr CL, Dean M, Clements ML: **Previving: How Unaffected Women with a**. *Health Commun* 2020, **35**(10):1256-1265.
35. Bancroft EK, Saya S, Brown E, Thomas S, Taylor N, Rothwell J, Pope J, Chamberlain A, Page E, Benafif S *et al*: **Psychosocial effects of whole-body MRI screening in adult high-risk pathogenic**. *J Med Genet* 2020, **57**(4):226-236.
36. Overbeek KA, Cahen DL, Kamps A, Konings ICAW, Harinck F, Kuenen MA, Koerkamp BG, Besselink MG, van Eijck CH, Wagner A *et al*: **Patient-reported burden of intensified surveillance and surgery in high-risk individuals under pancreatic cancer surveillance**. *Fam Cancer* 2020, **19**(3):247-258.
37. Rippinger N, Fischer C, Haun MW, Rhiem K, Grill S, Kiechle M, Cremer FW, Kast K, Nguyen HP, Ditsch N *et al*: **Cancer surveillance and distress among adult**

**pathogenic TP53 germline variant carriers in Germany: A multicenter feasibility and acceptance survey**. *Cancer* 2020, **126**(17):4032-4041.

1. Stracke C, Lemmen C, Rhiem K, Schmutzler R, Kautz-Freimuth S, Stock S: **Medical knowledge and information needs among women with pathogenic variants in moderate-risk genes for hereditary breast cancer attending genetic counseling at an academic hospital in Germany-A qualitative approach**. *J Genet Couns* 2022, **31**(3):698-712.
2. Kenen R, Ardern-Jones A, Eeles R: **Living with chronic risk: healthy women with a family history of breast/ovarian cancer** *Health, Risk & Society* 2003, **5**(3):315- 331.
3. Ando N, Iwamitsu Y, Kuranami M, Okazaki S, Yamamoto K, Watanabe M, Miyaoka H: **Concerns about inherited risk of breast cancer prior to diagnosis in Japanese patients with breast complaints**. *Fam Cancer* 2011, **10**(4):681-689.
4. Garland SN, Lounsberry J, Pelletier G, Bathe OF: **"How do you live without a stomach?": a multiple case study examination of total gastrectomy for palliation or prophylaxis**. *Palliat Support Care* 2011, **9**(3):305-313.
5. Heiniger L, Price MA, Charles M, Butow PN, Investigators kPGobotk: **Facilitators and Challenges in Psychosocial Adaptation to Being at Increased Familial Risk of Breast Cancer**. *J Genet Couns* 2015, **24**(6):890-907.
6. Dean M, Rauscher EA: **"It was an Emotional Baby": Previvors' Family Planning Decision-Making Styles about Hereditary Breast and Ovarian Cancer Risk**. *Journal of genetic counseling* 2017, **26**(6):1301-1313.
7. Meiser B, Quinn VF, Gleeson M, Kirk J, Tucker KM, Rahman B, Saunders C, Watts KJ, Peate M, Geelhoed E *et al*: **When knowledge of a heritable gene mutation comes out of the blue: treatment-focused genetic testing in women newly diagnosed with breast cancer**. *European journal of human genetics : EJHG* 2016, **24**(11):1517-1523.
8. Rauscher EA, Dean M: **"Take your time, then follow your heart:" Previvors' advice for communicating about family planning after testing positive for a BRCA genetic variant**. *Fam Syst Health* 2017, **35**(4):486-497.
9. Rietman AB, van Helden H, Both PH, Taal W, Legerstee JS, van Staa A, Moll HA, Oostenbrink R, van Eeghen AM: **Worries and needs of adults and parents of adults with neurofibromatosis type 1**. *Am J Med Genet A* 2018, **176**(5):1150-1160.
10. Young JL, Pantaleao A, Zaspel L, Bayer J, Peters JA, Khincha PP, Bremer RC, Loud JT, Greene MH, Achatz MI *et al*: **Couples coping with screening burden and diagnostic uncertainty in Li-Fraumeni syndrome: Connection versus independence**. *Journal of Psychosocial Oncology* 2019, **37**(2):178-193.
11. d'Agincourt-Canning L: **The effect of experiential knowledge on construction of risk perception in hereditary breast/ovarian cancer**. *J Genet Couns* 2005, **14**(1):55-69.
12. Bebbington Hatcher M, Fallowfield LJ: **A qualitative study looking at the psychosocial implications of bilateral prophylactic mastectomy**. *Breast* 2003, **12**(1):1-9.
13. Werner-Lin A, Rubin LR, Doyle M, Stern R, Savin K, Hurley K, Sagi M: **"My funky genetics": BRCA1/2 mutation carriers' understanding of genetic inheritance and reproductive merger in the context of new reprogenetic technologies**. *Fam Syst Health* 2012, **30**(2):166-180.
14. Heiniger L, Butow PN, Charles M, Price MA, Investigators kPGobotk: **Intuition versus cognition: a qualitative exploration of how women understand and manage their increased breast cancer risk**. *J Behav Med* 2015, **38**(5):727-739.
15. Michie S, McDonald V, Marteau T: **Understanding responses to predictive genetic testing: a grounded theory approach**. *Psychology & Health* 1996, **11**(4):455-470.
16. Scott S, Prior L, Wood F, Gray J: **Repositioning the patient: the implications of being 'at risk'**. *Soc Sci Med* 2005, **60**(8):1869-1879.
17. Palmquist AE, Koehly LM, Peterson SK, Shegog M, Vernon SW, Gritz ER: **"The cancer bond": exploring the formation of cancer risk perception in families with Lynch syndrome**. *J Genet Couns* 2010, **19**(5):473-486.
18. MacDonald DJ, Sarna L, Weitzel JN, Ferrell B: **Women's perceptions of the personal and family impact of genetic cancer risk assessment: focus group findings**. *J Genet Couns* 2010, **19**(2):148-160.
19. Campacci N, de Campos Reis Galvão H, Garcia LF, Ribeiro PC, Grasel RS, Goldim JR, Ashton-Prolla P, Palmero EI: **Genetic cancer risk assessment: A screenshot of the psychosocial profile of women at risk for hereditary breast and ovarian cancer syndrome**. *Psychooncology* 2020, **29**(4):681-687.
20. Bijlsma RM, Wessels H, Wouters RHP, May AM, Ausems M, Voest EE, Bredenoord AL: **Cancer patients' intentions towards receiving unsolicited genetic information obtained using next-generation sequencing**. *Fam Cancer* 2018, **17**(2):309-316.
21. Bradbury AR, Patrick-Miller LJ, Egleston BL, DiGiovanni L, Brower J, Harris D, Stevens EM, Maxwell KN, Kulkarni A, Chavez T *et al*: **Patient feedback and early outcome data with a novel tiered-binned model for multiplex breast cancer susceptibility testing**. *Genet Med* 2016, **18**(1):25-33.
22. Glassey R, O'Connor M, Ives A, Saunders C, kConFab I, O'Sullivan S, Hardcastle SJ: **Heightened perception of breast cancer risk in young women at risk of familial breast cancer**. *Fam Cancer* 2018, **17**(1):15-22.
23. Hallowell N, Badger S, Richardson S, Caldas C, Hardwick RH, Fitzgerald RC, Lawton J: **High-risk individuals' perceptions of reproductive genetic testing for CDH1 mutations**. *Fam Cancer* 2017, **16**(4):531-535.
24. Lieberman S, Lahad A, Tomer A, Cohen C, Levy-Lahad E, Raz A: **Population screening for BRCA1/BRCA2 mutations: lessons from qualitative analysis of the screening experience**. *Genet Med* 2017, **19**(6):628-634.
25. Makhnoon S, Garrett LT, Burke W, Bowen DJ, Shirts BH: **Experiences of patients seeking to participate in variant of uncertain significance reclassification research**. *J Community Genet* 2019, **10**(2):189-196.
26. McAllister MF, Evans DG, Ormiston W, Daly P: **Men in breast cancer families: a preliminary qualitative study of awareness and experience**. *J Med Genet* 1998, **35**(9):739-744.
27. Lodder L, Frets PG, Trijsburg RW, Tibben A, Meijers-Heijboer EJ, Duivenvoorden HJ, Wagner A, van Der Meer CA, Devilee P, Cornelisse CJ *et al*: **Men at risk of being a mutation carrier for hereditary breast/ovarian cancer: an exploration of attitudes and psychological functioning during genetic testing**. *Eur J Hum Genet* 2001, **9**(7):492-500.
28. d'Agincourt-Canning L: **A gift or a yoke? Women's and men's responses to genetic risk information from BRCA1 and BRCA2 testing**. *Clin Genet* 2006, **70**(6):462-472.
29. Hallowell N, Arden-Jones A, Eeles R, Foster C, Lucassen A, Moynihan C, Watson M: **Guilt, blame and responsibility: men's understanding of their role in the transmission of BRCA1/2 mutations within their family**. *Sociol Health Illn* 2006, **28**(7):969-988.
30. Mellon S, Gauthier J, Cichon M, Hammad A, Simon MS: **Knowledge, attitudes, and beliefs of Arab-American women regarding inherited cancer risk**. *J Genet Couns* 2013, **22**(2):268-276.
31. Campacci N, de Campos Reis Galvao H, Garcia LF, Ribeiro PC, Grasel RS, Goldim JR, Ashton-Prolla P, Palmero EI: **Genetic cancer risk assessment: A screenshot of the psychosocial profile of women at risk for hereditary breast and ovarian cancer syndrome**. *Psycho Oncology* 2020.
32. Vajen B, Rosset M, Wallaschek H, Baumann E, Schlegelberger B: **Psychological Distress and Coping Ability of Women at High Risk of Hereditary Breast and Ovarian Cancer before Undergoing Genetic Counseling-An Exploratory Study from Germany**. *Int J Environ Res Public Health* 2021, **18**(8).
33. Willis AM, Smith SK, Meiser B, James PA, Ballinger ML, Thomas DM, Yanes T, Young MA: **Influence of lived experience on risk perception among women who received a breast cancer polygenic risk score: 'Another piece of the pie'**. *J Genet Couns* 2021, **30**(3):849-860.
34. Dudok de Wit AC, Tibben A, Frets, PG, Meijers‐Heijboer, EJ, Devilee P, Niermeijer MF: **Males at–risk for the BRCA1**‐**1ene, the psychological impact**. *Psycho- Oncology* 1996, **5**(3):251-257.
35. Kenen R, Ardern-Jones A, Eeles R: **Family stories and the use of heuristics: women from suspected hereditary breast and ovarian cancer (HBOC) families**. *Sociol Health Illn* 2003, **25**(7):838-865.
36. Cohn WF, Fraser G, Jones SM, Miesfeldt S: **Perceptions of Cancer Risk, Risk Management and Family Issues: Views of Women At Risk for Hereditary Breast Cancer**. *Southern Nursing Research Society* 2008, **8**(3).
37. DiMillo J, Samson A, Thériault A, Lowry S, Corsini L, Verma S, Tomiak E: **Genetic testing: when prediction generates stigmatization**. *J Health Psychol* 2015, **20**(4):393-400.
38. Sankar P, Cho MK, Wolpe PR, Schairer C: **What is in a cause? Exploring the relationship between genetic cause and felt stigma**. *Genet Med* 2006, **8**(1):33-42.
39. Etchegary H, Miller F, deLaat S, Wilson B, Carroll J, Cappelli M: **Decision-making about inherited cancer risk: exploring dimensions of genetic responsibility**. *J Genet Couns* 2009, **18**(3):252-264.
40. Lynch HT, Watson P, Tinley S, Snyder C, Durham C, Lynch J, Kirnarsky Y, Serova O, Lenoir G, Lerman C *et al*: **An update on DNA-based BRCA1/BRCA2 genetic counseling in hereditary breast cancer**. *Cancer Genet Cytogenet* 1999, **109**(2):91- 98.
41. Hallowell N: **A qualitative study of the information needs of high-risk women undergoing prophylactic oophorectomy**. *Psychooncology* 2000, **9**(6):486-495.
42. Babb SA, Swisher EM, Heller HN, Whelan AJ, Mutch DG, Herzog TJ, Rader JS: **Qualitative Evaluation of Medical Information Processing Needs of 60 Women Choosing Ovarian Cancer Surveillance or Prophylactic Oophorectomy**. *J Genet Couns* 2002, **11**(2):81-96.
43. Polzer JM, Shawna L. Goel, Vivek: **Blood is thicker than water: Genetic testing as citizenship through familial obligation and the management of risk**. *Critical Public Health* 2002, **12**(2):153-168.
44. Hallowell N: **Negotiating present and future selves: managing the risk of hereditary ovarian cancer by prophylactic surgery**. *Health: An Interdisciplinary Journal for the Social Study of Health, Illness & Medicine* 2002.
45. Blandy C, Chabal F, Stoppa-Lyonnet D, Julian-Reynier C: **Testing participation in BRCA1/2-positive families: initiator role of index cases**. *Genet Test* 2003, **7**(3):225-233.
46. Kasparian NA, Meiser B, Butow PN, Job RF, Mann GJ: **Better the devil you know? High-risk individuals' anticipated psychological responses to genetic testing for melanoma susceptibility**. *J Genet Couns* 2006, **15**(6):433-447.
47. Mellon S, Berry-Bobovski L, Gold R, Levin N, Tainsky MA: **Communication and decision-making about seeking inherited cancer risk information: findings from female survivor-relative focus groups**. *Psychooncology* 2006, **15**(3):193-208.
48. McCullum M, Bottorff JL, Kelly M, Kieffer SA, Balneaves LG: **Time to decide about risk-reducing mastectomy: a case series of BRCA1/2 gene mutation carriers**. *BMC Womens Health* 2007, **7**:3.
49. Neuman HB, Robbins L, Duarte J, Charlson ME, Weiser MR, Guillem JG, Wong WD, Temple LK: **Risk-reducing surgery in FAP: role for surgeons beyond the incision**. *J Surg Oncol* 2010, **101**(7):570-576.
50. Ardern-Jones A, Kenen R, Lynch E, Doherty R, Eeles R: **Is no news good news? Inconclusive genetic test results in BRCA1 and BRCA2 from patients and professionals' perspectives**. *Hered Cancer Clin Pract* 2010, **8**(1):1.
51. Howard AF, Bottorff JL, Balneaves LG, Kim-Sing C: **Women's constructions of the 'right time' to consider decisions about risk-reducing mastectomy and risk- reducing oophorectomy**. *BMC Womens Health* 2010, **10**:24.
52. McQuirter M, Castiglia LL, Loiselle CG, Wong N: **Decision-making process of women carrying a BRCA1 or BRCA2 mutation who have chosen prophylactic mastectomy**. *Oncol Nurs Forum* 2010, **37**(3):313-320.
53. Wakefield CE, Ratnayake P, Meiser B, Suthers G, Price MA, Duffy J, Tucker K, KCNCfRiFBC: **"For all my family's sake, I should go and find out": an Australian report on genetic counseling and testing uptake in individuals at high risk of breast and/or ovarian cancer**. *Genet Test Mol Biomarkers* 2011, **15**(6):379-385.
54. Meiser B, Gleeson M, Kasparian N, Barlow-Stewart K, Ryan M, Watts K, Menon D, Mitchell G, Tucker K: **There is no decision to make: experiences and attitudes toward treatment-focused genetic testing among women diagnosed with ovarian cancer**. *Gynecol Oncol* 2012, **124**(1):153-157.
55. Griffith KA, Passmore SR, Smith D, Wenzel J: **African Americans with a family history of colorectal cancer: barriers and facilitators to screening**. *Oncol Nurs Forum* 2012, **39**(3):299-306.
56. Dean M, Fisher CL: **Uncertainty and previvors’ cancer risk management: Understanding the decision-making process**. *Journal of Applied Communication Research* 2019, **47**(4):460-483.
57. Garg R, Vogelgesang J, Kelly K: **Impact of Genetic Counseling and Testing on Altruistic Motivations to Test for BRCA1/2: a Longitudinal Study**. *Journal of Genetic Counseling* 2016, **25**(3):572-582.
58. Gietel-Habets JJG, de Die-Smulders CEM, Derks-Smeets IAP, Tibben A, Tjan- Heijnen VCG, van Golde R, Gomez-Garcia E, van Osch LADM: **Support needs of couples with hereditary breast and ovarian cancer during reproductive decision making**. *Psycho-Oncology* 2018, **27**(7):1795-1801.
59. Glassey R, O'Connor M, Ives A, Saunders C, Hardcastle SJ, Investigators k: **Influences on decision-making for young women undergoing bilateral prophylactic mastectomy**. *Patient Educ Couns* 2018, **101**(2):318-323.
60. Glassey R, O'Connor M, Ives A, Saunders C, kConFab I, O'Sullivan S, Hardcastle SJ: **Patients' perspectives and experiences concerning barriers to accessing information about bilateral prophylactic mastectomy**. *Breast* 2018, **40**:116-122.
61. Graham R, Owens M, Priest H, Hutton S: **Constructions of Decision Making for Risk-Reducing Mastectomy**. *Qualitative Health Research* 2018, **28**(10):1595-1609.
62. Grimmett C, Brooks C, Recio-Saucedo A, Armstrong A, Cutress RI, Gareth Evans D, Copson E, Turner L, Meiser B, Wakefield CE *et al*: **Development of Breast Cancer Choices: a decision support tool for young women with breast cancer deciding whether to have genetic testing for BRCA1/2 mutations**. *Support Care Cancer* 2019, **27**(1):297-309.
63. Hallowell N, Badger S, Richardson S, Caldas C, Hardwick RH, Fitzgerald RC, Lawton J: **An investigation of the factors effecting high-risk individuals' decision- making about prophylactic total gastrectomy and surveillance for hereditary diffuse gastric cancer (HDGC)**. *Familial Cancer* 2016, **15**(4):665-676.
64. Hamilton JG, Shuk E, Genoff MC, Rodriguez VM, Hay JL, Offit K, Robson ME: **Interest and Attitudes of Patients With Advanced Cancer With Regard to Secondary Germline Findings From Tumor Genomic Profiling**. *J Oncol Pract* 2017, **13**(7):e590-e601.
65. Kanga-Parabia A, Gaff C, Fl, er L, Jenkins M, Keogh LA: **Discussions about predictive genetic testing for Lynch syndrome: the role of health professionals and families in decisions to decline**. *Familial Cancer* 2018, **17**(4):547-555.
66. Lake PW, Kasting ML, Dean M, Fuzzell L, Hudson J, Carvajal R, Reed DR, Quinn GP, Vadaparampil ST: **Exploring patient and provider perspectives on the intersection between fertility, genetics, and family building**. *Supportive Care in Cancer* 2020.
67. Puski A, Hovick S, Senter L, Tol, AE: **Involvement and Influence of Healthcare Providers, Family Members, and Other Mutation Carriers in the Cancer Risk Management Decision-Making Process of BRCA1 and BRCA2 Mutation Carriers**. *Journal of Genetic Counseling* 2018, **27**(5):1291-1301.
68. Rauscher EA, Dean M, Campbell-Salome GM: **"I Am Uncertain About What My Uncertainty Even Is": Men's Uncertainty and Information Management of Their BRCA-Related Cancer Risks**. *Journal of Genetic Counseling* 2018, **27**(6):1417- 1427.
69. Rowland E, Plumridge G, Considine AM, Metcalfe A: **Preparing young people for future decision-making about cancer risk in families affected or at risk from hereditary breast cancer: A qualitative interview study**. *Eur J Oncol Nurs* 2016, **25**:9-15.
70. Shaw J, Bulsara C, Cohen PA, Gryta M, Nichols CB, Schofield L, O'Sullivan S, Pachter N, Hardcastle SJ: **Investigating barriers to genetic counseling and germline mutation testing in women with suspected hereditary breast and ovarian cancer syndrome and Lynch syndrome**. *Patient Education & Counseling* 2018, **101**(5):938-944.
71. Zimmermann BM, Shaw D, Heinimann K, Knabben L, Elger B, Kone I: **How the "control-fate continuum" helps explain the genetic testing decision-making process: a grounded theory study**. *European Journal of Human Genetics* 2020.
72. Sun S, Li ST, Ngeow J: **Factors shaping at-risk individuals' decisions to undergo genetic testing for cancer in Asia**. *Health Soc Care Community* 2020, **28**(5):1569- 1577.
73. Trister R, Jacobson M, Nguyen P, Sobel M, Allen L, Narod SA, Kotsopoulos J: **Patient reported experiences following laparoscopic prophylactic bilateral salpingo-oophorectomy or salpingectomy in an ambulatory care hospital**. *Fam Cancer* 2021, **20**(2):103-110.
74. Sa'at H, Lee YK, Yoon SY, Wong SW, Woo YL, Barlow-Stewart K, Mohd Taib NA: **The needs of Southeast Asian BRCA mutation carriers considering risk- reducing salpingo-oophorectomy: a qualitative study**. *Fam Cancer* 2022, **21**(1):21-33.
75. Zimmermann BM, Koné I, Shaw D, Elger B: **Autonomy and social influence in predictive genetic testing decision-making: A qualitative interview study**. *Bioethics* 2021, **35**(2):199-206.
76. Hoskins C, Tutty E, Purvis R, Shanahan M, Boussioutas A, Forrest L: **Young people's experiences of a CDH1 pathogenic variant: Decision-making about gastric cancer risk management**. *J Genet Couns* 2022, **31**(1):242-251.
77. Meadows RJ, Padamsee TJ: **Financial constraints on genetic counseling and further risk-management decisions among U.S. women at elevated breast cancer risk**. *J Genet Couns* 2021, **30**(5):1452-1467.
78. Gill G, Beard C, Storey K, Taylor S, Sexton A: **"It wasn't just for me": Motivations and implications of genetic testing for women at a low risk of hereditary breast and ovarian cancer syndrome**. *Psychooncology* 2020, **29**(8):1303-1311.
79. Scherr CL, Ramesh S, Getachew-Smith H, Kalke K, Ramsey K, Fischhoff B, Vadaparampil ST: **How patients deal with an ambiguous medical test: Decision- making after genetic testing**. *Patient Educ Couns* 2021, **104**(5):953-959.
80. Morand M, Roth M, Peterson SK, Bednar EM, Ramdaney A, Livingston JA, Yarbrough A, Corredor J: **Factors impacting adolescent and young adult cancer patients' decision to pursue genetic counseling and testing**. *Support Care Cancer* 2022, **30**(6):5481-5489.
81. Grosfeld FJ, Lips CJ, Ten Kroode HF, Beemer FA, Van Spijker HG, Brouwers- Smalbraak GJ: **Psychosocial consequences of DNA analysis for MEN type 2**. *Oncology (Williston Park)* 1996, **10**(2):141-146; discussion 146, 152, 157.
82. Lynch HT, Lemon SJ, Durham C, Tinley ST, Connolly C, Lynch JF, Surdam J, Orinion E, Slominski-Caster S, Watson P *et al*: **A descriptive study of BRCA1 testing and reactions to disclosure of test results**. *Cancer* 1997, **79**(11):2219-2228.
83. Appleton S, Fry A, Rees G, Rush R, Cull A: **Psychosocial effects of living with an increased risk of breast cancer: an exploratory study using telephone focus groups**. *Psychooncology* 2000, **9**(6):511-521.
84. Lloyd SM, Watson M, Oaker G, Sacks N, Querci della Rovere U, Gui G: **Understanding the experience of prophylactic bilateral mastectomy: a qualitative study of ten women**. *Psychooncology* 2000, **9**(6):473-485.
85. Lodder L, Frets PG, Trijsburg RW, Meijers-Heijboer EJ, Klijn JG, Duivenvoorden HJ, Tibben A, Wagner A, van der Meer CA, van den Ouweland AM *et al*: **Psychological impact of receiving a BRCA1/BRCA2 test result**. *Am J Med Genet* 2001, **98**(1):15- 24.
86. Bonadona V, Saltel P, Desseigne F, Mignotte H, Saurin JC, Wang Q, Sinilnikova O, Giraud S, Freyer G, Plauchu H *et al*: **Cancer patients who experienced diagnostic genetic testing for cancer susceptibility: reactions and behavior after the disclosure of a positive test result**. *Cancer Epidemiol Biomarkers Prev* 2002, **11**(1):97-104.
87. Lim J, Macluran M, Price M, Bennett B, Butow P, Group kP: **Short- and long-term impact of receiving genetic mutation results in women at increased risk for hereditary breast cancer**. *J Genet Couns* 2004, **13**(2):115-133.
88. Neary WJ, Stephens D, R.T. R, Evans G: **Psychosocial effects of neurofibromatosis type 2 (part 1): general effects**. *Audiological Medicine* 2006, **4**(4):202-210.
89. Neary WJ, Stephens D, Ramsden RT, Evans G: **Psychosocial effects of neurofibromatosis type 2 (Part 2): Effects on specific systems**. *Audiological Medicine* 2006, **4**(4):211-219.
90. Mireskandari S, Sangster J, Meiser B, Thewes B, Groombridge C, Spigelman A, Andrews L: **Psychosocial impact of familial adenomatous polyposis on young adults: a qualitative study**. *J Genet Couns* 2009, **18**(5):409-417.
91. Douglas HA, Hamilton RJ, Grubs RE: **The effect of BRCA gene testing on family relationships: A thematic analysis of qualitative interviews**. *J Genet Couns* 2009, **18**(5):418-435.
92. Stromsvik N, Raheim M, Oyen N, Engebretsen LF, Gjengedal E: **Stigmatization and male identity: Norwegian males' experience after identification as BRCA1/2 mutation carriers**. *J Genet Couns* 2010, **19**(4):360-370.
93. Bruwer Z, Futter M, Ramesar R: **Communicating cancer risk within an African context: experiences, disclosure patterns and uptake rates following genetic testing for Lynch syndrome**. *Patient Educ Couns* 2013, **92**(1):53-60.
94. Hallowell N, Alsop K, Gleeson M, Crook A, Plunkett L, Bowtell D, Mitchell G, Young MA, Group AOCS: **The responses of research participants and their next of kin to receiving feedback of genetic test results following participation in the Australian Ovarian Cancer Study**. *Genet Med* 2013, **15**(6):458-465.
95. Caiata-Zufferey M: **Genetically at-risk status and individual agency. A qualitative study on asymptomatic women living with genetic risk of breast/ovarian cancer**. *Soc Sci Med* 2015, **132**:141-148.
96. Becker M: **Women's descriptions six months post notification of positive BRCA 1/2 genetic mutations**. *THESIS.* ProQuest Information & Learning; 2018.
97. Dean M, Scherr CL, Clements M, Koruo R, Martinez J, Ross A: **"When information is not enough": A model for understanding BRCA-positive previvors' information needs regarding hereditary breast and ovarian cancer risk**. *Patient Education & Counseling* 2017, **100**(9):1738-1743.
98. Hallowell N, Badger S, Richardson S, Caldas C, Hardwick RH, Fitzgerald RC, Lawton J: **High-risk individuals' perceptions of reproductive genetic testing for CDH1 mutations**. *Familial Cancer* 2017, **16**(4):531-535.
99. Hesse-Biber S: **Gender differences in psychosocial and medical outcomes stemming from testing positive for the BRCA1/2 genetic mutation for breast cancer: An explanatory sequential mixed methods study**. *Journal of Mixed Methods Research* 2018, **12**(3):280-304.
100. Kajula O, Kuismin O, Kyngäs H: **Identification as a mutation carrier and effects on life according to experiences of Finnish male BRCA1/2 mutation carriers**. *Journal of Genetic Counseling* 2018, **27**(4):874-884.
101. Mahat-Shamir M, Possick C: **The experience of women carriers of BRCA mutations following risk-reducing surgery: A cultural perspective**. *Health Care for Women International* 2017, **38**(4):344-360.
102. McBride KA, Ballinger ML, Schlub TE, Young MA, Tattersall MHN, Kirk J, Eeles R, Killick E, Walker LG, Shanley S *et al*: **Psychosocial morbidity in TP53 mutation carriers: is whole-body cancer screening beneficial?** *Familial Cancer* 2017, **16**(3):423-432.
103. Zhu X, Leof ER, Rabe KG, McCormick JB, Petersen GM, Radecki Breitkopf C: **Psychological Impact of Learning CDKN2A Variant Status as a Genetic Research Result**. *Public Health Genomics* 2018, **21**(3):154-163.
104. Padmanabhan H, Hassan NT, Wong SW, Lee YQ, Lim J, Hasan SN, Yip CH, Teo SH, Thong MK, Mohd Taib NA *et al*: **Psychosocial outcome and health behaviour intent of breast cancer patients with BRCA1/2 and PALB2 pathogenic variants unselected by a priori risk**. *PLoS One* 2022, **17**(2):e0263675.
105. Dwyer AA, Hesse-Biber S, Shea H, Zeng Z, Yi S: **Coping response and family communication of cancer risk in men harboring a BRCA mutation: A mixed methods study**. *Psychooncology* 2022, **31**(3):486-495.
106. Forrest LE, Forbes Shepherd R, Tutty E, Pearce A, Campbell I, Devereux L, Trainer AH, James PA, Young MA: **The Clinical and Psychosocial Outcomes for Women Who Received Unexpected Clinically Actionable Germline Information Identified through Research: An Exploratory Sequential Mixed-Methods Comparative Study**. *J Pers Med* 2022, **12**(7).
107. Lynch HT, Watson P, Shaw TG, Lynch JF, Harty AE, Franklin BA, Kapler CR, Tinley ST, Liu B, Lerman C: **Clinical impact of molecular genetic diagnosis, genetic counseling, and management of hereditary cancer. Part II: Hereditary nonpolyposis colorectal carcinoma as a model**. *Cancer* 1999, **86**(11 Suppl):2457- 2463.
108. Lodder LN, Frets PG, Trijsburg RW, Meijers-Heijboer EJ, Klijn JG, Duivenvoorden HJ, Tibben A, Wagner A, van der Meer CA, Devilee P *et al*: **Presymptomatic testing for BRCA1 and BRCA2: how distressing are the pre-test weeks? Rotterdam/Leiden Genetics Working Group**. *J Med Genet* 1999, **36**(12):906-913.
109. Reeve J, Owens RG, Winship IM: **Psychological impact of predictive testing for colonic cancer**. *J Health Psychol* 2000, **5**(1):99-108.
110. Hallowell N, Foster C, Ardern-Jones A, Eeles R, Murday V, Watson M: **Genetic testing for women previously diagnosed with breast/ovarian cancer: examining the impact of BRCA1 and BRCA2 mutation searching**. *Genet Test* 2002, **6**(2):79- 87.
111. Hallowell N, Foster C, Eeles R, Ardern-Jones A, Watson M: **Accommodating risk: responses to BRCA1/2 genetic testing of women who have had cancer**. *Soc Sci Med* 2004, **59**(3):553-565.
112. Claes E, Evers-Kiebooms G, Boogaerts A, Decruyenaere M, Denayer L, Legius E: **Diagnostic genetic testing for hereditary breast and ovarian cancer in cancer patients: women's looking back on the pre-test period and a psychological evaluation**. *Genet Test* 2004, **8**(1):13-21.
113. Eisenbruch M, Yeo SS, Meiser B, Goldstein D, Tucker K, Barlow-Stewart K: **Optimising clinical practice in cancer genetics with cultural competence: lessons to be learned from ethnographic research with Chinese-Australians**. *Soc Sci Med* 2004, **59**(2):235-248.
114. Kausmeyer DT, Lengerich EJ, Kluhsman BC, Morrone D, Harper GR, Baker MJ: **A survey of patients' experiences with the cancer genetic counseling process: recommendations for cancer genetics programs**. *J Genet Couns* 2006, **15**(6):409- 431.
115. Grant AM, van Teijlingen ER, Forrest-Keenan K, Torrance N, Wilson BJ, Haites NE: **Does breast cancer genetic counselling meet women's expectations? A qualitative study**. *Critical Public Health* 2006, **16**(4):281-293.
116. Phelps C, Bennett P, Iredale R, Anstey S, Gray J: **The development of a distraction-based coping intervention for women waiting for genetic risk information: a phase 1 qualitative study**. *Psychooncology* 2006, **15**(2):169-173.
117. Ford ME, Alford SH, Britton D, McClary B, Gordon HS: **Factors influencing perceptions of breast cancer genetic counseling among women in an urban health care system**. *J Genet Couns* 2007, **16**(6):735-753.
118. Phelps C, Wood F, Bennett P, Brain K, Gray J: **Knowledge and expectations of women undergoing cancer genetic risk assessment: a qualitative analysis of free-text questionnaire comments**. *J Genet Couns* 2007, **16**(4):505-514.
119. Hamilton RJ, Bowers BJ: **The Theory of Genetic Vulnerability: a Roy model exemplar**. *Nurs Sci Q* 2007, **20**(3):254-264.
120. Metcalfe A, Werrett J, Burgess L, Clifford C: **Psychosocial impact of the lack of information given at referral about familial risk for cancer**. *Psychooncology* 2007, **16**(5):458-465.
121. Duncan RE, Gillam L, Savulescu J, Williamson R, Rogers JG, Delatycki MB: **The challenge of developmentally appropriate care: predictive genetic testing in young people for familial adenomatous polyposis**. *Fam Cancer* 2010, **9**(1):27-35.
122. Buckmaster AM, Gallagher P: **Experiences of and perspectives on genetic testing for breast/ovarian cancer in and outside of the customary clinical setting**. *Psychol Health* 2010, **25**(9):1041-1059.
123. Gammon AD, Rothwell E, Simmons R, Lowery JT, Ballinger L, Hill DA, Boucher KM, Kinney AY: **Awareness and preferences regarding BRCA1/2 genetic counseling and testing among Latinas and non-Latina white women at increased risk for hereditary breast and ovarian cancer**. *J Genet Couns* 2011, **20**(6):625-638.
124. Macdonald DJ, Deri J, Ricker C, Perez MA, Ogaz R, Feldman N, Viveros LA, Paz B, Weitzel JN, Blazer KR: **Closing the loop: an interactive action-research conference format for delivering updated medical information while eliciting Latina patient/family experiences and psychosocial needs post-genetic cancer risk assessment**. *Fam Cancer* 2012, **11**(3):449-458.
125. Zilliacus E, Meiser B, Gleeson M, Watts K, Tucker K, Lobb EA, Mitchell G: **Are we being overly cautious? A qualitative inquiry into the experiences and perceptions of treatment-focused germline BRCA genetic testing amongst women recently diagnosed with breast cancer**. *Support Care Cancer* 2012, **20**(11):2949-2958.
126. Aspinwall LG, Taber JM, Leaf SL, Kohlmann W, Leachman SA: **Genetic testing for hereditary melanoma and pancreatic cancer: a longitudinal study of psychological outcome**. *Psychooncology* 2013, **22**(2):276-289.
127. Francke U, Dijamco C, Kiefer AK, Eriksson N, Moiseff B, Tung JY, Mountain JL: **Dealing with the unexpected: consumer responses to direct-access BRCA mutation testing**. *PeerJ* 2013, **1**:e8.
128. MacLeod R, Beach A, Henriques S, Knopp J, Nelson K, Kerzin-Storrar L: **Experiences of predictive testing in young people at risk of Huntington's disease, familial cardiomyopathy or hereditary breast and ovarian cancer**. *Eur J Hum Genet* 2014, **22**(3):396-401.
129. Joseph M, Rab F, Panabaker K, Nisker J: **Feelings of Women With Strong Family Histories Who Subsequent to Their Breast Cancer Diagnosis Tested BRCA Positive**. *Int J Gynecol Cancer* 2015, **25**(4):584-592.
130. Augestad MT, Høberg-Vetti H, Bjorvatn C, Sekse RJT: **Identifying needs: A**

**qualitative study of women’s experiences regarding rapid genetic testing for hereditary breast and ovarian cancer in the DNA BONus study**. *Journal of Genetic Counseling* 2017, **26**(1):182-189.

1. Brunstrom K, Murray A, McAllister M: **Experiences of Women Who Underwent Predictive BRCA 1/2 Mutation Testing Before the Age of 30**. *Journal of Genetic Counseling* 2016, **25**(1):90-100.
2. Fisher CL, Roccotagliata T, Rising CJ, Kissane DW, Glogowski EA, Bylund CL: **"I Don't Want to Be an Ostrich": Managing Mothers' Uncertainty during BRCA1/2 Genetic Counseling**. *Journal of Genetic Counseling* 2017, **26**(3):455-468.
3. Glassey R, Hardcastle SJ, O'Connor M, Ives A, Saunders C: **Perceived influence of psychological consultation on psychological well**‐**being, body image, and intimacy following bilateral prophylactic mastectomy: A qualitative analysis**. *Psycho-Oncology* 2018, **27**(2):633-639.
4. Godino L, Jackson L, Turchetti D, Hennessy C, Skirton H: **Decision making and experiences of young adults undergoing presymptomatic genetic testing for familial cancer: a longitudinal grounded theory study**. *European Journal of Human Genetics* 2018, **26**(1):44-53.
5. McLeavy L, Rahman B, Kristeleit R, Ledermann J, Lockley M, McCormack M, Mould T, Side L, Lanceley A: **Mainstreamed genetic testing in ovarian cancer: patient experience of the testing process**. *International Journal of Gynecological Cancer* 2020, **30**(2):221-226.
6. Medendorp NM, Hillen MA, Murugesu L, Aalfs CM, Stiggelbout AM, Smets EMA: **Uncertainty in consultations about genetic testing for cancer: an explorative observational study**. *Patient Education & Counseling* 2018, **101**(12):2083-2089.
7. Rajpal N, Munoz J, Peshkin BN, Graves KD: **Insights into BRCA1/2 Genetic Counseling from Ethnically Diverse Latina Breast Cancer Survivors**. *Journal of Genetic Counseling* 2017, **26**(6):1221-1237.
8. Rasmussen V, Forbes Shepherd R, Forrest LE, James PA, Young MA: **Men's experiences of recontact about a potential increased risk of prostate cancer due to Lynch Syndrome: "Just another straw on the stack"**. *Journal of genetic counseling* 2019, **28**(4):750-759.
9. Shipman H, Flynn S, MacDonald-Smith CF, Brenton J, Crawford R, Tischkowitz M, Hulbert-Williams NJ: **Universal BRCA1/BRCA2 testing for ovarian cancer patients is welcomed, but with care: How women and staff contextualize experiences of expanded access**. *Journal of Genetic Counseling* 2017, **26**(6):1280-1291.
10. Bartley N, Best M, Butow P: **Pursuing germline genome sequencing to reduce illness uncertainty may involve additional uncertainties for cancer patients: A mixed-methods study**. *J Genet Couns* 2021, **30**(4):1143-1155.
11. Blomen CL, Pott A, Volk AE, Budäus L, Witzel I: **Communication processes about predictive genetic testing within high-risk breast cancer families: a two-phase study design**. *Sci Rep* 2021, **11**(1):20178.
12. Rolle L, Zayhowski K, Koeller D, Chiluiza D, Carmichael N: **Transgender patients' perspectives on their cancer genetic counseling experiences**. *J Genet Couns* 2022, **31**(3):781-791.
13. Matsukawa M, Torishima M, Satoh C, Honda S, Kosugi S: **Japanese women's reasons for accompaniment status to hereditary breast and ovarian cancer- focused genetic counseling**. *J Genet Couns* 2022, **31**(2):497-509.
14. Gómez-Trillos S, Sheppard VB, Graves KD, Song M, Anderson L, Ostrove N, Lopez K, Campos C, Gonzalez N, Hurtado-de-Mendoza A: **Latinas' knowledge of and experiences with genetic cancer risk assessment: Barriers and facilitators**. *J Genet Couns* 2020, **29**(4):505-517.
15. Bartley N, Napier CE, Butt Z, Schlub TE, Best MC, Biesecker BB, Ballinger ML, Butow P: **Cancer Patient Experience of Uncertainty While Waiting for Genome Sequencing Results**. *Front Psychol* 2021, **12**:647502.
16. Lee DS, Meiser B, Mariapun S, Hassan T, Yip CH, Mohd Taib NA, Teo SH, Thong MK, Yoon SY: **Communication about positive BRCA1 and BRCA2 genetic test results and uptake of testing in relatives in a diverse Asian setting**. *J Genet Couns* 2021, **30**(3):720-729.
17. Underhill-Blazey M, Blonquist T, Chittenden A, Pozzar R, Nayak M, Lansang K, Hong F, Garber J, Stopfer JE: **Informing models of cancer genetics care in the era of multigene panel testing with patient-led recommendations**. *J Genet Couns* 2021, **30**(1):268-282.
18. Battistuzzi L, Franiuk M, Kasparian N, Rania N, Migliorini L, Varesco L: **A qualitative study on decision-making about BRCA1/2 testing in Italian women**. *Eur J Cancer Care (Engl)* 2019, **28**(5):e13083.
19. Lohn Z, Fok A, Richardson M, Derocher H, Mung SW, Nuk J, Yuson J, Jevon M, A Schrader K, Sun S: **Large-scale group genetic counseling: Evaluation of a novel service delivery model in a Canadian hereditary cancer clinic**. *J Genet Couns* 2022, **31**(2):459-469.
20. Kasparian NA, Meiser B, Butow PN, Soames Job RF, Mann GJ: **Anticipated uptake of genetic testing for familial melanoma in an Australian sample: An exploratory study**. *Psychooncology* 2007, **16**(1):69-78.
21. Foster C, Watson M, Moynihan C, Arden-Jones A, Eeles R: **Juggling roles and expectations: dilemmas faced by women talking to relatives about cancer and genetic testing**. *Psychology & Health* 2004, **19**(4).
22. Hallowell N, Ardern-Jones A, Eeles R, Foster C, Lucassen A, Moynihan C, Watson M: **Communication about genetic testing in families of male BRCA1/2 carriers and non-carriers: patterns, priorities and problems**. *Clin Genet* 2005, **67**(6):492-502.
23. McCann S, MacAuley D, Barnett Y, Bunting B, Bradley A, Jeffers L, Morrison PJ: **Family communication, genetic testing and colonoscopy screening in hereditary non-polyposis colon cancer: a qualitative study**. *Psychooncology* 2009, **18**(11):1208-1215.
24. Maddock C, Schrijvers D, Turco M, Marotti L, Sullivan R: **To know or not to know? Not the only question in familial breast cancer risk communication**. *Ecancermedicalscience* 2011, **5**:239.
25. Fisher CL, Maloney E, Glogowski E, Hurley K, Edgerson S, Lichtenthal WG, Kissane D, Bylund C: **Talking about familial breast cancer risk: topics and strategies to enhance mother-daughter interactions**. *Qual Health Res* 2014, **24**(4):517-535.
26. Chopra I, Kelly KM: **Cancer Risk Information Sharing: The Experience of Individuals Receiving Genetic Counseling for BRCA1/2 Mutations**. *Journal of Health Communication* 2017, **22**(2):143-152.
27. Derbez B: **Is there a "right time" for bad news? Kairos in familial communication on hereditary breast and ovarian cancer risk**. *Social Science & Medicine* 2018, **202**:13-19.
28. Joseph G, Pasick R, Schillinger D, Luce J, Guerra C, Cheng J: **Information Mismatch: Cancer Risk Counseling with Diverse Underserved Patients**. *Journal of Genetic Counseling* 2017, **26**(5):1090-1104.
29. Young J, Pantaleao A, Green MH, Loud JT, Khincha PP, Bremer RC, Achatz MI, Werner-Lin A: **Conference Abstract Couples coping with Li-Fraumeni syndrome: Anticipatory loss and the role of the support person**. *Psycho-Oncology* 2018, **27**:75-76.
30. Hamilton RJ, Bowers BJ, Williams JK: **Disclosing genetic test results to family members**. *J Nurs Scholarsh* 2005, **37**(1):18-24.
31. Cypowyj C, Eisinger F, Huiart L, Sobol H, Morin M, Julian-Reynier C: **Subjective interpretation of inconclusive BRCA1/2 cancer genetic test results and transmission of information to the relatives**. *Psychooncology* 2009, **18**(2):209- 215.
32. Peshkin BN, Demarco TA, Tercyak KP: **On the development of a decision support intervention for mothers undergoing BRCA1/2 cancer genetic testing regarding communicating test results to their children**. *Fam Cancer* 2010, **9**(1):89-97.
33. Dancyger C, Wiseman M, Jacobs C, Smith JA, Wallace M, Michie S: **Communicating BRCA1/2 genetic test results within the family: a qualitative analysis**. *Psychol Health* 2011, **26**(8):1018-1035.
34. Maloney E, Edgerson S, Robson M, Offit K, Brown R, Bylund C, Kissane DW: **What women with breast cancer discuss with clinicians about risk for their adolescent daughters**. *J Psychosoc Oncol* 2012, **30**(4):484-502.
35. Patenaude AF, Tung N, Ryan PD, Ellisen LW, Hewitt L, Schneider KA, Tercyak KP, Aldridge J, Garber JE: **Young adult daughters of BRCA1/2 positive mothers: what do they know about hereditary cancer and how much do they worry?** *Psychooncology* 2013, **22**(9):2024-2031.
36. Farkas Patenaude A, DeMarco TA, Peshkin BN, Valdimarsdottir H, Garber JE, Schneider KA, Hewitt L, Hamilton J, Tercyak KP: **Talking to children about maternal BRCA1/2 genetic test results: a qualitative study of parental perceptions and advice**. *J Genet Couns* 2013, **22**(3):303-314.
37. Baars J, Ausems M, Riel E, Kars M, Bleiker E: **Communication Between Breast Cancer Patients Who Received Inconclusive Genetic Test Results and Their Daughters and Sisters Years After Testing**. *Journal of Genetic Counseling* 2016, **25**(3):461-471.
38. Dean M, Rauscher EA: **Men's and Women's Approaches to Disclosure About BRCA-Related Cancer Risks and Family Planning Decision-Making**. *Qualitative Health Research* 2018, **28**(14):2155-2168.
39. Li ST, Sun S, Lie D, Met-Domestici M, Courtney E, Menon S, Lim GH, Ngeow J: **Factors influencing the decision to share cancer genetic results among family members: An in-depth interview study of women in an Asian setting**. *Psycho- Oncology* 2018, **27**(3):998-1004.
40. Seenandan-Sookdeo KA, Hack TF, Lobchuk M, Murphy L, Marles S: **Parental Decision Making Regarding the Disclosure or Nondisclosure of a Mutation- Positive BRCA1/2 Test Result to Minors**. *Oncol Nurs Forum* 2016, **43**(3):330-341.
41. Bradbury AR, Patrick-Miller L, Pawlowski K, Ibe CN, Cummings SA, Hlubocky F, Olopade OI, Daugherty CK: **Learning of your parent's BRCA mutation during adolescence or early adulthood: a study of offspring experiences**. *Psychooncology* 2009, **18**(2):200-208.
42. Crotser CB, Dickerson SS: **Learning about a twist in the road: perspectives of at- risk relatives learning of potential for cancer**. *Oncol Nurs Forum* 2010, **37**(6):723- 733.
43. Crotser CB, Dickerson SS: **Women receiving news of a family BRCA1/2 mutation: messages of fear and empowerment**. *J Nurs Scholarsh* 2010, **42**(4):367-378.
44. Himes DO, Gibbons DK, Birmingham WC, Beckstr, L. R, Gammon A, Kinney AY, Clayton MF: **Female family members lack understanding of indeterminate negative brca1/2 test results shared by probands**. *Journal of Genetic Counseling* 2019.
45. Foster C, Watson M, Moynihan C, Ardern-Jones A, Eeles R: **Genetic testing for breast and ovarian cancer predisposition: cancer burden and responsibility**. *J Health Psychol* 2002, **7**(4):469-484.
46. Ormondroyd E, Moynihan C, Watson M, Foster C, Davolls S, Ardern-Jones A, Eeles R: **Disclosure of genetics research results after the death of the patient participant: a qualitative study of the impact on relatives**. *J Genet Couns* 2007, **16**(4):527-538.
47. Werner-Lin AV: **Danger zones: risk perceptions of young women from families with hereditary breast and ovarian cancer**. *Fam Process* 2007, **46**(3):335-349.
48. Kasparian NA, Butow PN, Meiser B, Mann GJ: **High- and average-risk individuals' beliefs about, and perceptions of, malignant melanoma: an Australian perspective**. *Psychooncology* 2008, **17**(3):270-279.
49. Benjamin C, Flynn M, Hallett C, Ellis I, Booth K: **The use of the life course paradigm and life course charts to explore referral for family history of breast cancer**. *Int J Nurs Stud* 2008, **45**(1):95-109.
50. Dagan E, Goldblatt H: **The twilight zone between health and sickness: a qualitative exploration with asymptomatic BRCA1 and 2 mutation carriers**. *Women Health* 2009, **49**(4):263-279.
51. Maheu C: **Implications of living with a strong family history of breast cancer**.

*Can J Nurs Res* 2009, **41**(2):100-112.

1. Campbell-Salome G, Rauscher EA: **Family storytelling about hereditary cancer: Framing shared understandings of risk**. *J Genet Couns* 2020, **29**(6):936-948.
2. Hughes L, Phelps C: **"The bigger the network the bigger the bowl of cherries...": exploring the acceptability of, and preferences for, an ongoing support network for known BRCA 1 and BRCA 2 mutation carriers**. *J Genet Couns* 2010, **19**(5):487-496.
3. Miller FA, Carroll JC, Wilson BJ, Bytautas JP, Allanson J, Cappelli M, de Laat S, Saibil F: **The primary care physician role in cancer genetics: a qualitative study of patient experience**. *Fam Pract* 2010, **27**(5):563-569.
4. Dean M, Rauscher E, Gomez E, Fischer C: **Expectations versus reality: The impact of men's expectancy violations in conversations with healthcare providers about BRCA-related cancer risks**. *Patient Education & Counseling* 2019, **102**(9):1650-1655.
5. Fadda M, Chappuis PO, Katapodi MC, Pagani O, Monnerat C, Membrez V, Unger S, Caiata Zufferey M: **Physicians communicating with women at genetic risk of breast and ovarian cancer: Are we in the middle of the ford between contradictory messages and unshared decision making?** *PLoS One* 2020, **15**(10):e0240054.
6. Zimmermann BM, Fanderl J, Koné I, Rabaglio M, Bürki N, Shaw D, Elger B: **Examining information-seeking behavior in genetic testing for cancer predisposition: A qualitative interview study**. *Patient Educ Couns* 2021, **104**(2):257-264.
